# Supplementary material for: Targeting cardiomyocyte ADAM10 ectodomain shedding promotes survival early after myocardial infarction
Source: Nat Commun. 2022 Dec 10;13:7648. doi: 10.1038/s41467-022-35331-0 (PMC9741599; doi:10.1038/s41467-022-35331-0)
Supplement: Supplementary file 1 — Supplementary Information [file 41467_2022_35331_MOESM1_ESM.pdf]

SUPPLEMENTARY FIGURES

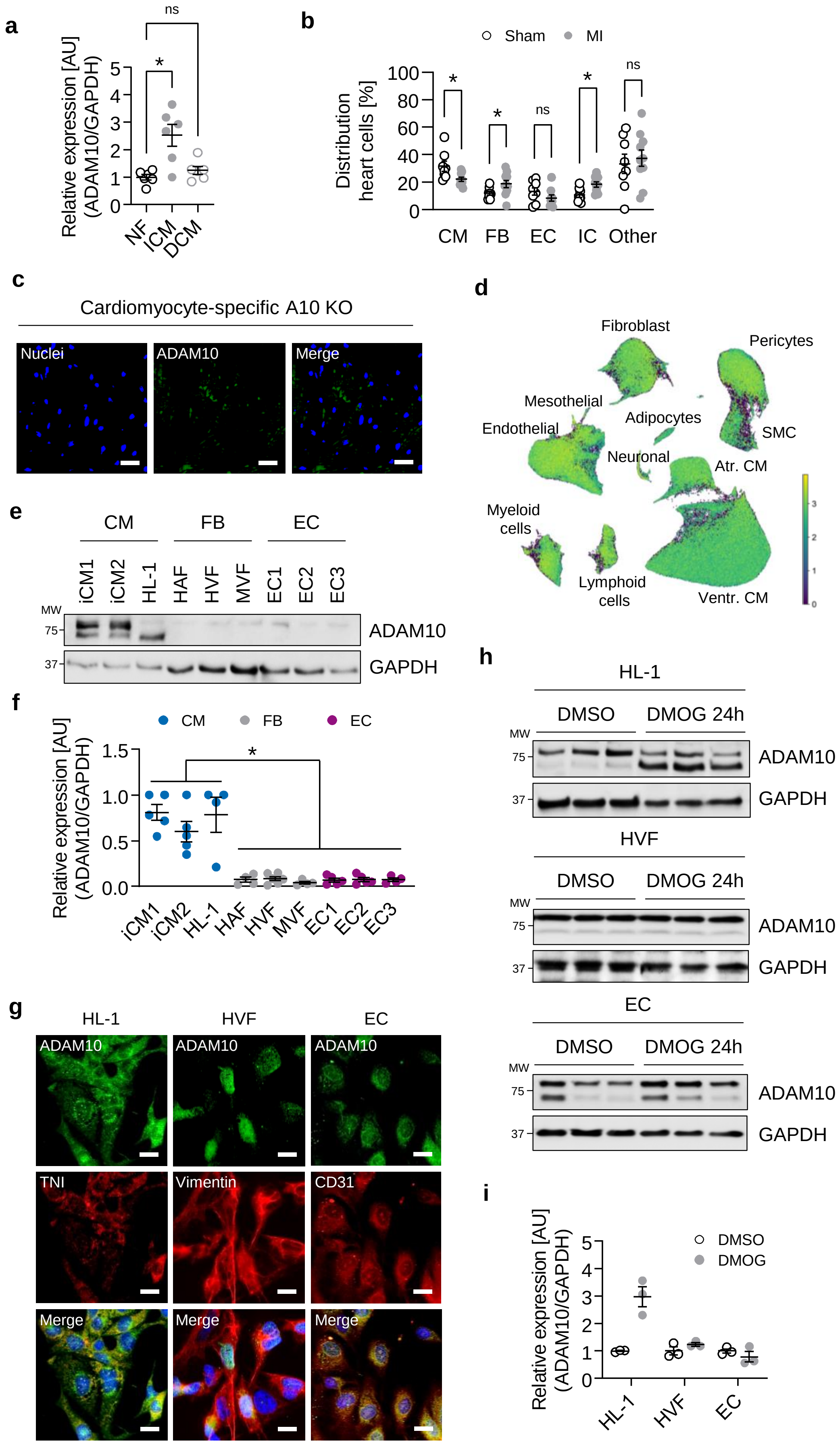

Supplementary Fig 1. ADAM10 expression in cardiomyocytes. a Quantification of ADAM10 in heart tissue lysates from patients with ischemic cardiomyopathy (ICM) and dilated cardiomyopathy (DCM) versus non-failing controls (NF) (n = 6, mean ± SEM, *P<0.05, one-way ANOVA with Tukey's posttest). **b** Quantification of the fraction of indicated cell types in stained heart tissue sections of sham-operated (Sham) and LAD-ligated (MI) mice 14 d after surgery. n = 4 per group, mean ± SEM, ns = not significant, *P<0.05, two-tailed Mann-Whitney test. **c** Immunofluorescence images and of ADAM10 (A10) stained heart tissue sections of 6-month-old mice with cardiomyocyte-specific ADAM10 knockout. Nuclei are stained with DAPI. Representative images are shown. Scale bar, 25 µm. n = 3 per group. **d** Uniform manifold approximation and projection (UMAP) showing ADAM10 expression in 11 cardiac cell types. Publicly available single cell sequencing data of the "Heart Cell Atlas" were analyzed. SMC, smooth muscle cells. Atr. CM, atrial cardiomyocytes. Ventr. CM, ventricular cardiomyocytes. **e** Western blot analysis of ADAM10 in human iPSC-derived cardiomyocytes (iCM1-2), mouse cardiomyocytes (HL-1), human atrial fibroblasts (HAF), human ventricular fibroblasts (HVF), primary mouse ventricular fibroblasts (MVF) and independent pools of human umbilical vein endothelial cells (EC1-3) (n = 5). CM, cardiomyocytes. FB, fibroblasts. EC, endothelial cells. **f** Quantification of ADAM10 protein expression in human iPSC-derived cardiomyocytes (iCM1-2), mouse cardiomyocytes (HL-1), human atrial fibroblasts (HAF), human ventricular fibroblasts (HVF), primary mouse ventricular fibroblasts (MVF) and independent pools of human umbilical vein endothelial cells (EC1-3) (n = 5, mean ± SEM, **P<0.001, Kruskal-Wallis test). CM, cardiomyocytes. FB, fibroblasts. EC, endothelial cells. **g** Immunofluorescence images of ADAM10, TNI, Vimentin and CD31 stained mouse cardiomyocytes (HL-1), human ventricular fibroblasts (HVF) and human umbilical vein endothelial cells (EC) (n = 3). Representative images are shown. Scale bar, 20 µm. **h** Western blot analysis and **i** quantification of pro- (pADAM10) and mature ADAM10 (mADAM10) in DMSO and DMOG (1 mM) treated mouse cardiomyocytes (HL-1), human ventricular fibroblasts (HVF) and human umbilical vein endothelial cells (EC) (n = 3, mean ± SEM, two-tailed Mann-Whitney test with Dunn's posttest). Source data are provided as a Source Data file.

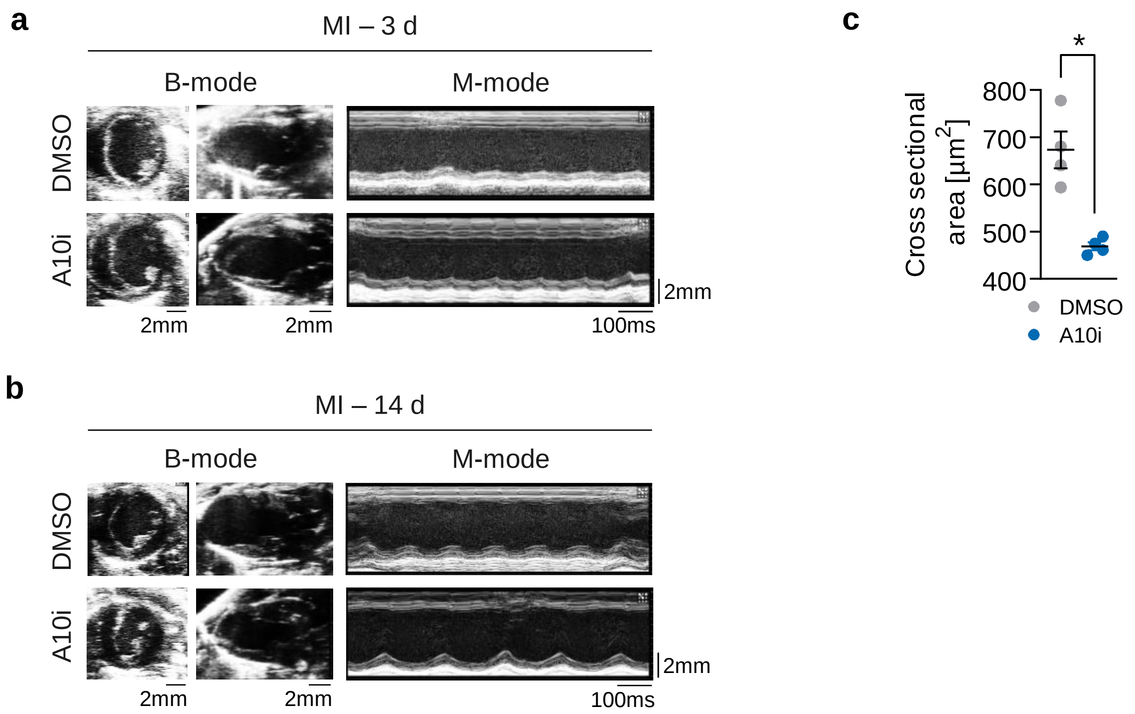

Supplementary Fig. 2. Pharmacological ADAM10 inhibition augments cardiac function after experimental infarction. a-b Representative end-systolic B-mode and M-mode echocardiograms of GI254023X (A10i) and DMSO treated mice 3 and 14 d after myocardial infarction. **c** Quantification of TNI stained heart tissue sections of GI254023X (A10i) and DMSO treated mice 28 d after myocardial infarction (n = 4, mean ± SEM, *P<0.05, two-tailed Mann-Whitney test). Source data are provided as a Source Data file.

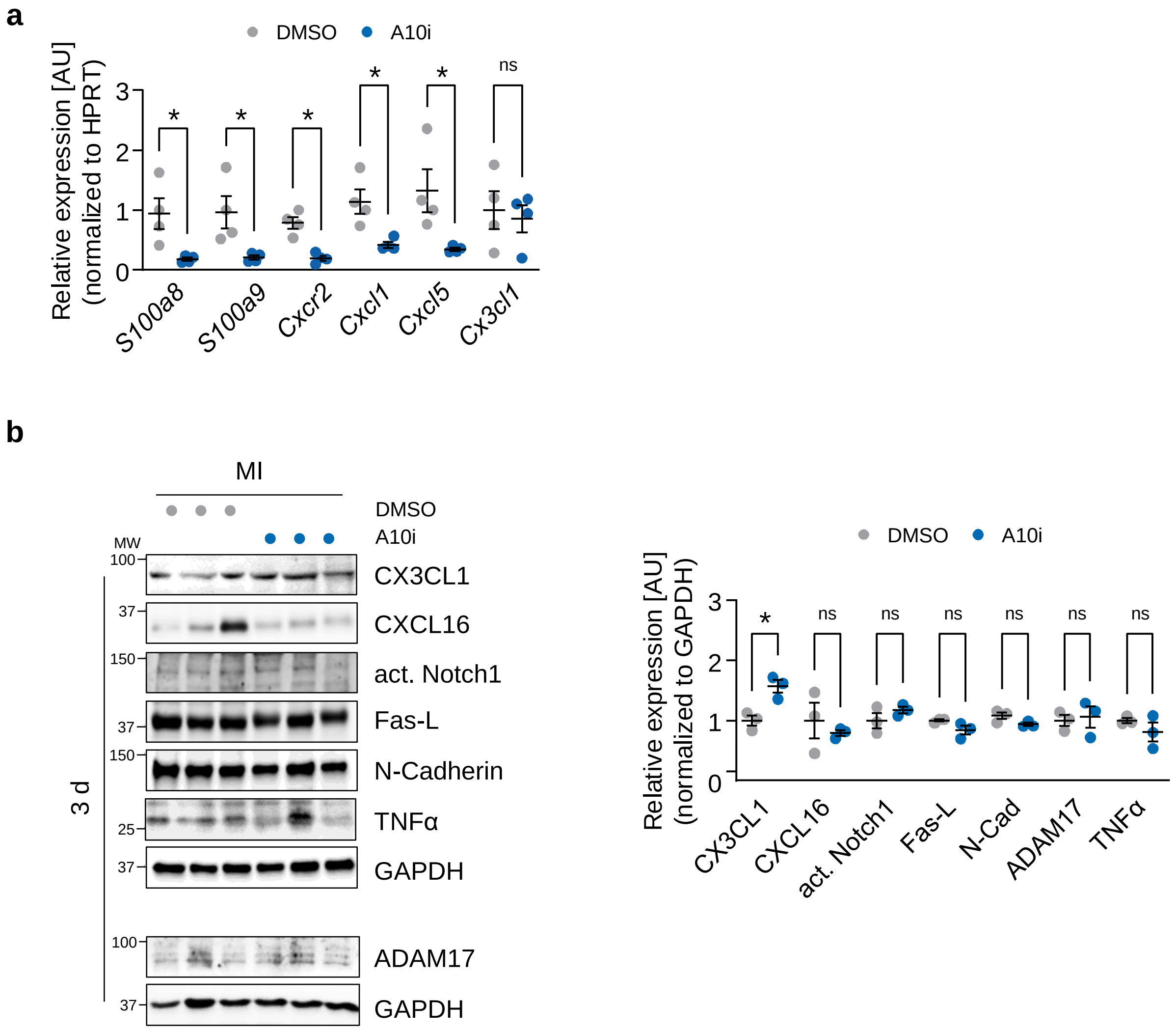

Supplementary Fig. 3. Pharmacological ADAM10 inhibitor reduces chemotaxis-associated gene expression and CX3CL1 shedding. a Quantitative real-time PCR of significantly downregulated neutrophil chemotaxis-associated genes in heart tissue of A10i (n= 5) and DMSO (n = 4) treated mice 3 d after MI (mean ± SEM, *P<0.05, two-tailed Mann-Whitney test with Dunn's posttest). **b** Western blot analysis and quantification of indicated proteins in heart tissue lysates of GI254023X (A10i) and DMSO treated mice 3 d after myocardial infarction (n = 3, mean ± SEM, ns = not significant, *P<0.05, two-tailed Mann-Whitney test). Source data are provided as a Source Data file.

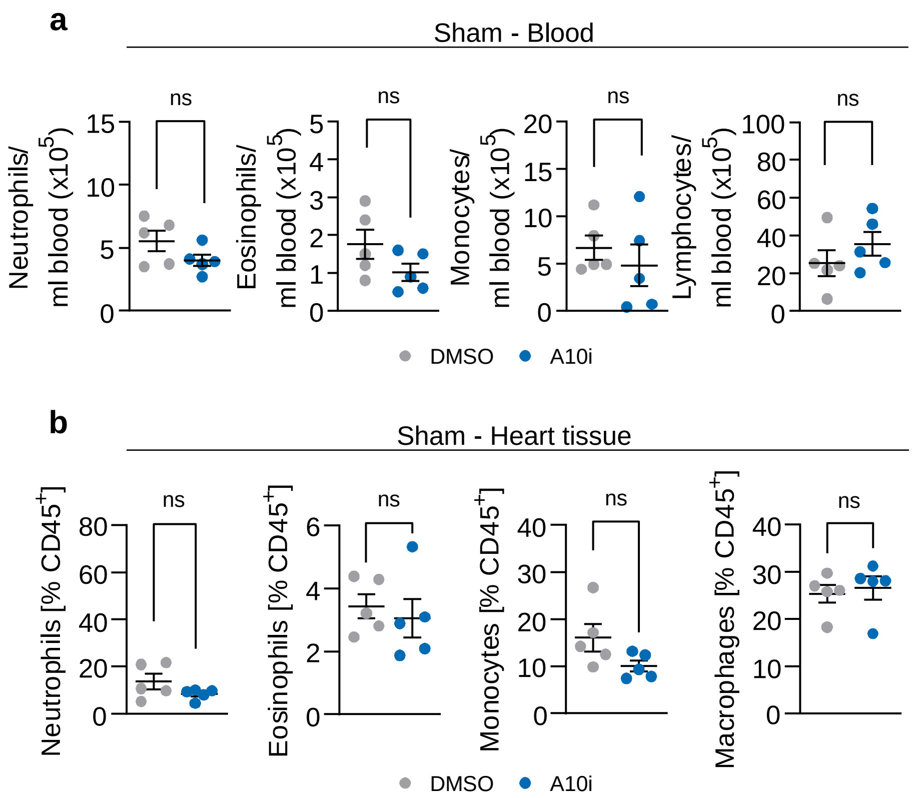

Supplementary Fig. 4. ADAM10 inhibition does not affect leukocyte bone marrow egress and heart tissue infiltration in sham-operated mice. a Analysis of leukocyte counts in blood samples of GI254023X (A10i, n = 5) and DMSO (n = 5) treated mice 3 d after infarction (mean ± SEM, ns = not significant, **P<0.01, two-tailed t test). **b** Quantification of neutrophils ($CD45^+$, $CD11b^+$, $Ly6G^+$), eosinophils ($CD45^+$, $CD11b^+$, $Ly6G^-$, $SigF^+$), monocytes ($CD45^+$, $CD11b^+$, $Ly6G^-$, $SigF^-$, $F4/80^-$, $Ly6C^+$) and macrophages ($CD45^+$, $CD11b^+$, $Ly6G^-$, $SigF^-$, $F4/80^+$) in heart tissue of A10i (n = 5) and DMSO (n = 5) treated mice 3 d after sham operation (mean ± SEM, ns = not significant, two-tailed t test). Source data are provided as a Source Data file.

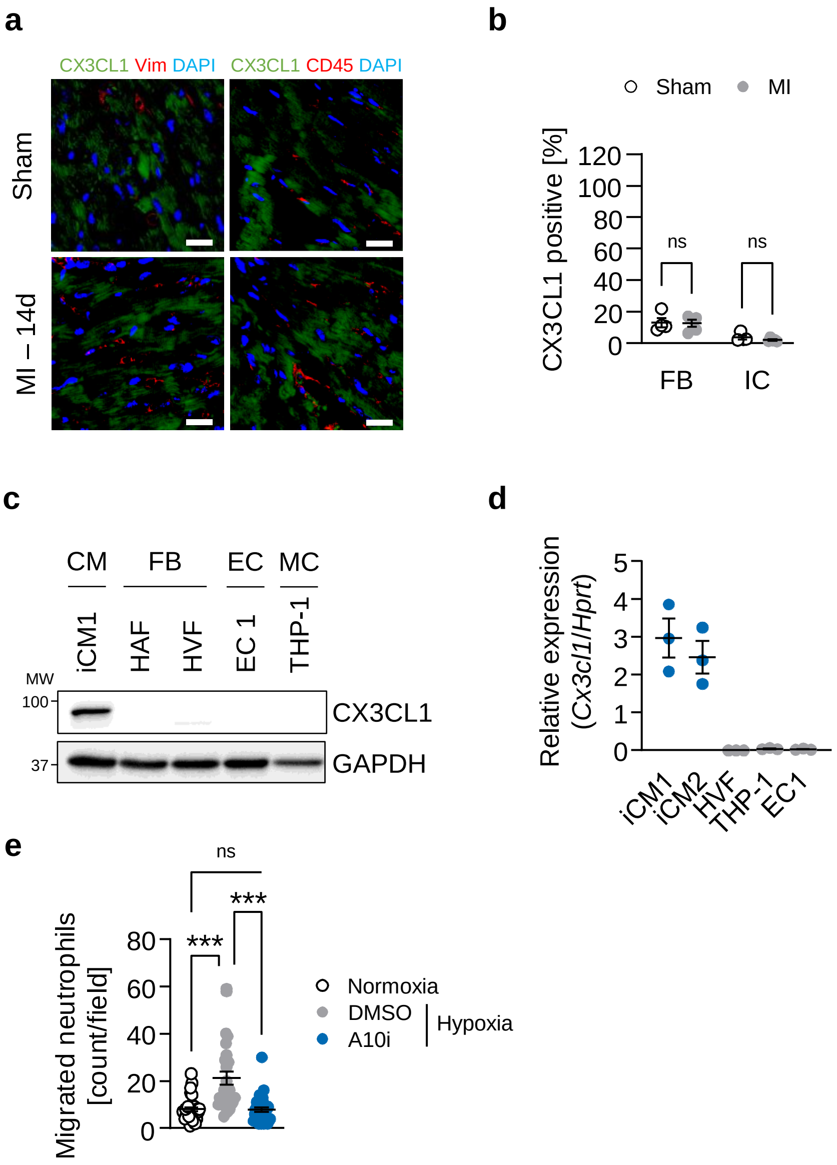

Supplementary Fig. 5. CX3CL1 is expressed in cardiomyocytes and regulates neutrophil transmigration upon hypoxia. a Immunofluorescence images and **b** quantification of CX3CL1, Vimentin (Vim - fibroblasts) and CD45 (immune cells) stained heart tissue sections of sham-operated (Sham) and LAD-ligated (MI) mice 14 d after surgery. Nuclei are stained with DAPI. Representative images are shown. Scale bar, 20 µm. n = 4 per group, mean ± SEM, ns = not significant, two-tailed Mann-Whitney test with Dunn's posttest. **c** Western blot analysis of CX3CL1 expression in human iPSC-derived cardiomyocytes (iCM1), human ventricular fibroblasts (HVF), human umbilical vein endothelial cells (EC1) and human monocytic cells (THP-1). Representative western blots (n = 3 per group) are shown. CM, cardiomyocytes. FB, fibroblasts. EC, endothelial cells. MC, monocytes. **d** Quantification of *CX3CL1* mRNA expression in human iPSC-derived cardiomyocytes (iCM1-2), human ventricular fibroblasts (HVF), human umbilical vein endothelial cells (EC1) and human monocytic cells (THP-1) (n = 3, mean ± SEM, Kruskal-Wallis test). CM, cardiomyocytes. FB, fibroblasts. EC, endothelial cells. MC, monocytes. **e** Transwell migration assays of bone marrow derived neutrophils using the supernatants of normoxic as well as A10i and DMSO treated hypoxic HL-1 cells as chemoattractant (n = 3 per group, mean ± SEM, ns = not significant, ***P<0.001, one-way ANOVA with Tukey's posttest). Source data are provided as a Source Data file.

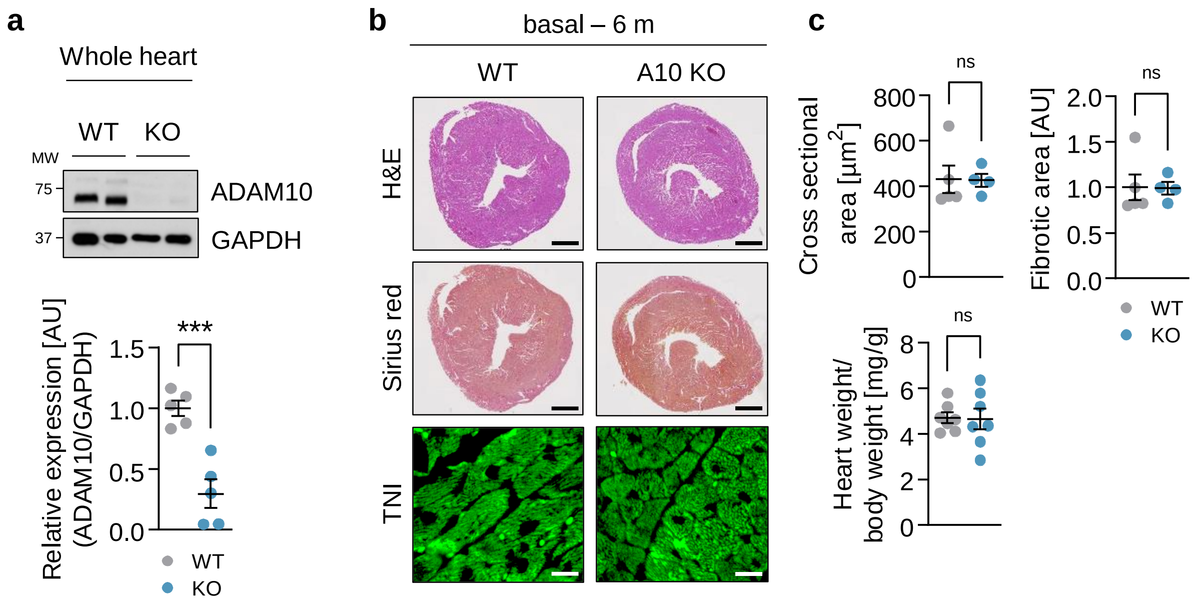

Supplementary Fig. 6. Basal characterization of cardiomyocyte-specific ADAM10 knockout mice. a Western blot analysis and quantification of ADAM10 expression in heart tissue samples of 8-week-old ADAM10fl/fl (WT) and cardiomyocyte-specific (αMHC-Cre) ADAM10 knockout (KO) mice (n = 5, mean ± SEM, ***P<0.01, two-tailed t test). **b** Representative images and **c** quantification of H&E (n = 6), picro sirius red (n = 6) and TNI stained (n = 4) heart tissue sections of 8-week-old ADAM10 WT and cardiomyocyte-specific ADAM10 KO mice (mean ± SEM, ns = not significant, two-tailed Mann-Whitney test). Source data are provided as a Source Data file.

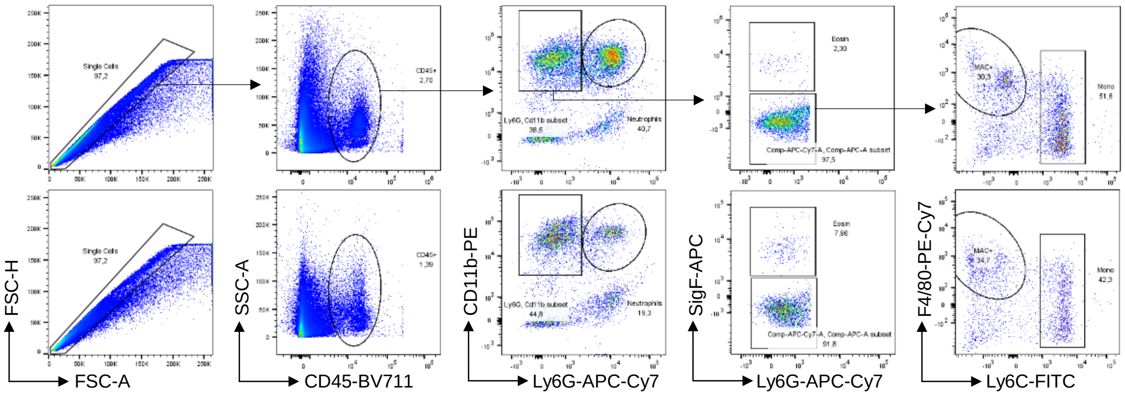

Supplementary Fig. 7. Cardiomyocyte-specific ADAM10 knockout reduces neutrophil bone marrow egress and heart tissue infiltration following myocardial infarction. Gating strategy for identification of neutrophils (CD45$^+$, CD11b$^+$, Ly6G$^+$), eosinophils (CD45$^+$, CD11b$^+$, Ly6G$^-$, SigF$^+$), monocytes (CD45$^+$, CD11b$^+$, Ly6G$^-$, SigF$^-$, F4/80$^-$, Ly6C$^+$) and macrophages (CD45$^+$, CD11b$^+$, Ly6G$^-$, SigF$^-$, F4/80$^+$) in the infarcted area/ infarct border zone of ADAM10 KO (n = 7) and WT (n = 8) treated mice 3 d after infarction. Source data are provided as a Source Data file.

Supplementary Table 1. Primer sequences. Fwd, forward primer. Rev, reverse primer.

Species	Gene	Sequences
Homo sapiens	*ADAM10*	Fwd: 5’-AGCAACATCTGGGGACAAAC-3’
		Rev: 5’- CCCAGGTTTCAGTTTGCATT-3’
	CX3CL1	Fwd: 5’- ACAGCACCACGGTGTGACGAAA-3’
		Rev: 5’- AACAGCCTGTGCTGTCTCGTCT-3’
	HPRT1	Fwd: 5’- CCTGGCGTCGTGATTAGTG-3’
		Rev: 5’- ACAGAGGGCTACAATGTGATGG-3’
	NPPA	Fwd: 5’- CACCGTGAGCTTCCTCCTTT-3’
		Rev: 5’- CCAAATGGTCCAGCAAATTCTTG-3’
	NPPB	Fwd: 5’- CTTTCCTGGGAGGTCGTTCC-3’
		Rev: 5’- GTTGCGCTGCTCCTGTAAC-3’
Mus musculus	*Adam10*	Fwd: 5’- ATGGTGTTGCCGACAGTGTTA-3’
		Rev: 5’- GTTTGGCACGCTGGTGTTTTT-3’
	Cx3cl1	Fwd: 5’- GAGCATCACTGACATCTACCTCC-3’
		Rev: 5’- AGAAGGCAGTCGTGAGCTTGCA-3’
	Cxcl1	Fwd: 5’- TCCAGAGCTTGAAGGTGTTGCC-3’
		Rev: 5’- AACCAAGGGAGCTTCAGGGTCA-3’
	Cxcl3	Fwd: 5’- TGAGACCATCCAGAGCTTGACG-3’
		Rev: 5’- CCTTGGGGGTTGAGGCAAACTT-3’
	Cxcl5	Fwd: 5’- CCGCTGGCATTTCTGTTGCTGT-3’
		Rev: 5’- CAGGGATCACCTCCAAATTAGCG-3’
	Cxcr2	Fwd: 5’- CTCTATTCTGCCAGATGCTGTCC-3’
		Rev: 5’- ACAAGGCTCAGCAGAGTCACCA-3’
	Hprt1	Fwd: 5’- TTGGGCTTACCTCACTGCTT-3’
		Rev: 5’- CATCATCGCTAATCACGACGC-3’
	S100a8	Fwd: 5’- CAAGGAAATCACCATGCCCTCTA-3’
		Rev: 5’- ACCATCGCAAGGAACTCCTCGA-3’
	S100a9	Fwd: 5’- TGGTGGAAGCACAGTTGGCAAC-3’
		Rev: 5’- CAGCATCATACACTCCTCAAAGC-3’
