# Supplementary material for: Targeting cardiomyocyte ADAM10 ectodomain shedding promotes survival early after myocardial infarction
Source: Nat Commun. 2022 Dec 10;13:7648. doi: 10.1038/s41467-022-35331-0 (PMC9741599; doi:10.1038/s41467-022-35331-0)
Supplement: Supplementary file 3 — Reporting Summary [file 41467_2022_35331_MOESM3_ESM.pdf]

nature portfolio

Corresponding author(s): Ali El-Armouche

Last updated by author(s): Nov 6, 2022

Reporting Summary

Nature Portfolio wishes to improve the reproducibility of the work that we publish. This form provides structure for consistency and transparency in reporting. For further information on Nature Portfolio policies, see our Editorial Policies and the Editorial Policy Checklist.

Statistics

For all statistical analyses, confirm that the following items are present in the figure legend, table legend, main text, or Methods section.

n/a	Confirmed	
☐	☒	The exact sample size (*n*) for each experimental group/condition, given as a discrete number and unit of measurement
☐	☒	A statement on whether measurements were taken from distinct samples or whether the same sample was measured repeatedly
☐	☒	The statistical test(s) used AND whether they are one- or two-sided<br>*Only common tests should be described solely by name; describe more complex techniques in the Methods section.*
☐	☒	A description of all covariates tested
☐	☒	A description of any assumptions or corrections, such as tests of normality and adjustment for multiple comparisons
☐	☒	A full description of the statistical parameters including central tendency (e.g. means) or other basic estimates (e.g. regression coefficient) AND variation (e.g. standard deviation) or associated estimates of uncertainty (e.g. confidence intervals)
☐	☒	For null hypothesis testing, the test statistic (e.g. *F*, *t*, *r*) with confidence intervals, effect sizes, degrees of freedom and *P* value noted<br>*Give P values as exact values whenever suitable.*
☒	☐	For Bayesian analysis, information on the choice of priors and Markov chain Monte Carlo settings
☒	☐	For hierarchical and complex designs, identification of the appropriate level for tests and full reporting of outcomes
☐	☒	Estimates of effect sizes (e.g. Cohen's *d*, Pearson's *r*), indicating how they were calculated

Our web collection on statistics for biologists contains articles on many of the points above.

Software and code

Policy information about availability of computer code

Data collection	FusionCapt Advance software (Vilber); Vevo 3100 (VisualSonics); Gene5 v2.09 (BioTek); BZ-X Viewer v1.03.00.05 (Keyence); CFX manager 3.1 (BioRad); RNA-SeQC (1.1.8); GSNAP (v2020-12-16); featureCounts (v2.0.1); R packages (v.4.0.4) DESeq2 R package (v1.30.1) and IHW (1.18.0); BDFACS Diva software version 8.0.2 (BD Biosciences)
Data analysis	Scanpy 1.8.2; CellProfiler 4; Fiji; Vevo 2.1.0 (VisualSonics); CFX manager 3.1 for 2-ΔΔCt calculations (BioRad); Enrichr version March 2021 (https://maayanlab.cloud/Enrichr); GSEA tool v4.1.0 (Broad Institute); FlowJo version 10.8.0 (BD Life Sciences); GraphPad Prism version 9.4.1 (Dotmatics); Excel version 2207 (Microsoft)

For manuscripts utilizing custom algorithms or software that are central to the research but not yet described in published literature, software must be made available to editors and reviewers. We strongly encourage code deposition in a community repository (e.g. GitHub). See the Nature Portfolio guidelines for submitting code & software for further information.

Data

Policy information about availability of data

All manuscripts must include a data availability statement. This statement should provide the following information, where applicable:

- Accession codes, unique identifiers, or web links for publicly available datasets
- A description of any restrictions on data availability
- For clinical datasets or third party data, please ensure that the statement adheres to our policy

The annotated human single cell gene expression data to evaluate ADAM10 expression were retrieved from the Heart Cell Atlas [https://cellgeni.cog.sanger.ac.uk/heartcellatlas/data/global_raw.h5ad]. The mus musculus reference genome mm10 (RefSeq assembly accession code: GCF_000001635.20) [https://www.ncbi.nlm.nih.gov/assembly/GCF_000001635.20] was used to align RNA sequencing reads. The RNA sequencing data generated in this study have been

deposited in the Gene Expression Omnibus (GEO) under accession code GSE217268 [https://www.ncbi.nlm.nih.gov/geo/query/acc.cgi?acc=GSE217268]. Source data are provided with this paper.

Field-specific reporting

Please select the one below that is the best fit for your research. If you are not sure, read the appropriate sections before making your selection.

- [x] Life sciences
- [ ] Behavioural & social sciences
- [ ] Ecological, evolutionary & environmental sciences

For a reference copy of the document with all sections, see nature.com/documents/nr-reporting-summary-flat.pdf

Life sciences study design

All studies must disclose on these points even when the disclosure is negative.

Sample size	Sample size for each experiment is indicated in the legend. For animal experiments (ADAM10 inhibitor) statistical tests were used to pre-determine sample size, or sample size was chosen based on animal availability (ADAM10 WT / KO). For cell culture and biochemical experiments no statistical tests were used to pre-determine sample size, but sample size was chosen based on previous experiments and comparable studies in literature, which is most optimal to generate statistically significant results. For adequate power, we generally chose a sample size of n = 3–6.
Data exclusions	To ensure comparability throughout the study, mice were excluded only if successful LAD-ligation could not be determined by echocardiography and histology.
Replication	Sample size and number of experimental replicates are displayed in the figure captions.
Randomization	Mice were assigned randomly to the experimental groups. For experiments other than those involving mice, samples were randomly allocated into experimental groups.
Blinding	Surgery, treatment and echocardiography were performed in a blinded fashion.

Reporting for specific materials, systems and methods

We require information from authors about some types of materials, experimental systems and methods used in many studies. Here, indicate whether each material, system or method listed is relevant to your study. If you are not sure if a list item applies to your research, read the appropriate section before selecting a response.

Materials & experimental systems

n/a	Involved in the study	
[ ]	[x]	Antibodies
[ ]	[x]	Eukaryotic cell lines
[x]	[ ]	Palaeontology and archaeology
[ ]	[x]	Animals and other organisms
[ ]	[x]	Human research participants
[x]	[ ]	Clinical data
[x]	[ ]	Dual use research of concern

Methods

n/a	Involved in the study	
[x]	[ ]	ChIP-seq
[ ]	[x]	Flow cytometry
[x]	[ ]	MRI-based neuroimaging

Antibodies

Antibodies used

Antibody/ Supplier / catalog # / clone/ lot #:

ADAM8 Polyclonal Antibody rabbit/ Proteintech/ 23778-1-AP
Recombinant Anti-ADAM10 Antibody rabbit/ Abcam/ ab124695/ EPR5622/ GR3380698-1
Anti-ADAM10 Antibody rabbit/ Millipore/ AB19026/ 2382103
ADAM12 Polyclonal antibody rabbit/ Proteintech/ 14139-1-AP
ADAM17 Polyclonal antibody rabbit/ Proteintech/ 24620-1-AP
ADAM19 Antibody rabbit/ Novus biologicals/ NBP1-69364/ QC19590-42849
Purified Rat Anti-Mouse CD31/ BD Biosciences/ 550274/ MEC 13.3 (RUO)/ 9259767
Purified Mouse Anti-Human CD45/ BD Biosciences/ 555480/ HI30 (RUO)/ 2039584
Mouse CXCL16 Antibody/ R&D Systems/ MAB503/ Clone # 142417/ FHY022010C
Mouse CX3CL1/Fractalkine Chemokine Domain Antibody/ R&D Systems/ AF472/ / CKQ0821011
FAS-L (C-20)/ Santa Cruz/ sc-957/ C-20/ E1708
GAPDH mouse/ Santa Cruz/ sc-365062/ G-9/ I2320
IL-1β (3A6) Mouse mAb/ Cell Signaling/ #12242/ 3A6/ 1/Ref 10/2019

Purified Mouse Anti-N-Cadherin/ BD Biosciences/ 610921/ 32/N-Cadherin/ 4114645
Anti-activated Notch1 antibody/ Abcam/ ab8925/ /GR218543-26
TNF-α (D2D4) XP® Rabbit mAb/ Cell Signaling/ #11948/ D2D4/ 7
Anti-Cardiac Troponin I (cTnI) Antibody mouse/ Millipore/ MAB3150/ clone 284/ NG1866953
Anti-Cardiac Troponin T (cTnT) antibody/ Abcam/ ab8295/ 1C11/ 3322222-1
Purified Mouse Anti-Vimentin/ BD Biosciences/ BD550513/ RV202 (RUO)/ 1274497
TruStain FcX™ PLUS (anti-mouse CD16/32) Antibody / Biolegend/ 156604/ S17011E/ B330293
Brilliant Violet 711™ anti-mouse CD45 Antibody/ Biolegend/ 103147/ 30-F11/ B339309
APC/Cyanine7 anti-mouse Ly-6G Antibody/ BioLegend/ 127624/ 1A8/ B339475
CD11b Monoclonal Antibody (M1/70), PE, eBioscience™/ invitrogen Thermo Fisher /12-0112-82/ M1_70/ 2296701
FITC Rat Anti-Mouse Ly-6C/ BD Biosciences/ 553104/ AL-21/ 1172671
Alexa Fluor® 647 Rat Anti-Mouse Siglec-F/ BD Bioscience/ 562680 /E50-2440/ 1179948
F4/80 Monoclonal Antibody (BM8), PE-Cyanine7, eBioscience™ / invitrogen _ Thermo Fisher/ 25-4801-82/ BM8/ 2338684
TruStain FcX™ PLUS (anti-mouse CD16/32) Antibody / Biolegend/ 156604/ S17011E/ B330293
Brilliant Violet 711™ anti-mouse CD45 Antibody/ Biolegend/ 103147/ 30-F11/ B339309
APC/Cyanine7 anti-mouse Ly-6G Antibody/ BioLegend/ 127624/ 1A8/ B339475
CD11b Monoclonal Antibody (M1/70), PE, eBioscience™/ invitrogen Thermo Fisher /12-0112-82/ M1_70/ 2296701
FITC Rat Anti-Mouse Ly-6C/ BD Biosciences/ 553104/ AL-21/ 1172671
Alexa Fluor® 647 Rat Anti-Mouse Siglec-F/ BD Bioscience/ 562680 /E50-2440/ 1179948
F4/80 Monoclonal Antibody (BM8), PE-Cyanine7, eBioscience™ / invitrogen _ Thermo Fisher/ 25-4801-82/ BM8/ 2338684
Alexa Fluor 488-coupled goat anti-rabbit / ThermoFisher Scientific / A11008 / / 2284595
Alexa Fluor 546-couppled goat anti-mouse / ThermoFisher Scientific / A-11003 / / 2273717
CF 594-coupled goat anti-rat / Sigma-Aldrich / SAB4600111 / / 10C0208

Validation

Validation statements available from manufacturers (antibody /supplier name/ catalog# / validation statement):

ADAM8 Polyclonal antibody rabbit/ Proteintech/ 23778-1-AP/ https://www.ptglab.com/products/ADAM8-Antibody-23778-1-AP.htm
Recombinant Anti-ADAM10 antibody rabbit/ Abcam/ ab124695/ https://www.abcam.com/adam10-antibody-epr5622-ab124695.html

Anti-ADAM10 Antibody rabbit/ Millipore/ AB19026/ https://www.merckmillipore.com/DE/de/product/Anti-ADAM-10-Antibody-CT,MM_NF-AB19026?ReferrerURL=https%3A%2F%2Fwww.google.com%2F

ADAM12 Polyclonal antibody rabbit/ Proteintech/ 14139-1-AP/ https://www.ptglab.com/products/ADAM12-Antibody-14139-1-AP.htm

ADAM17 Polyclonal antibody rabbit/ Proteintech/ 24620-1-AP/ https://www.ptglab.com/products/ADAM17-Antibody-24620-1-AP.htm

ADAM19 Antibody rabbit/ Novus biologicals/ NBP1-69364/ https://www.novusbio.com/products/adam19-antibody_nbp1-69364
Purified Rat Anti-Mouse CD31/ BD Biosciences/ 550274/ https://www.bdbiosciences.com/en-ca/products/reagents/flow-cytometry-reagents/research-reagents/single-color-antibodies-ruo/purified-rat-anti-mouse-cd31.550274

Purified Mouse Anti-Human CD45/ BD Biosciences/ 555480/ https://www.bdbiosciences.com/en-us/products/reagents/flow-cytometry-reagents/research-reagents/single-color-antibodies-ruo/purified-mouse-anti-human-cd45.555480

Mouse CXCL16 Antibody/ R&D Systems/ MAB503/ https://www.rndsystems.com/products/mouse-cxcl16-antibody-142417_mab503

Mouse CX3CL1/Fractalkine Chemokine Domain Antibody/ R&D Systems/ AF472/ https://www.rndsystems.com/products/mouse-cx3cl1-fractalkine-chemokine-domain-antibody_af472

FAS-L (C-20)/ Santa Cruz/ sc-957/ https://www.scbt.com/de/p/fas-l-antibody-c-20

GAPDH Antikörper mouse/ Santa Cruz/ SC-365062/ https://www.scbt.com/de/p/gapdh-antibody-g-9

IL-1β (3A6) Mouse mAb/ Cell Signaling/ #12242/ https://www.cellsignal.com/products/primary-antibodies/il-1b-3a6-mouse-mab/12242

Purified Mouse Anti-N-Cadherin/ BD Biosciences/ 610921/ https://www.bdbiosciences.com/en-us/products/reagents/microscopy-imaging-reagents/immunofluorescence-reagents/purified-mouse-anti-n-cadherin.610921

Anti-activated Notch1 antibody/ Abcam/ ab8925/ https://www.abcam.com/activated-notch1-antibody-ab8925.html

TNF-α (D2D4) XP® Rabbit mAb/ Cell Signaling/ #11948/ https://www.cellsignal.com/products/primary-antibodies/tnf-a-d2d4-xp-rabbit-mab-mouse-specific/11948

Anti-Cardiac Troponin I Antibody mouse/ Millipore/ MAB3150/ https://www.merckmillipore.com/DE/de/product/Anti-Cardiac-Troponin-I-a.a.-41-49-clone-284-19C7-1mg-KC,MM_NF-MAB3150-KC

Anti-Cardiac Troponin T antibody/ Abcam/ ab8295/ 1C11/ https://www.abcam.com/cardiac-Troponin-T-antibody-1C11-ab8295.html

Purified Mouse Anti-Vimentin/ BD Biosciences/ 550513/ https://www.bdbiosciences.com/en-de/products/reagents/microscopy-imaging-reagents/purified-mouse-anti-vimentin.550513

TruStain FcX™ PLUS (anti-mouse CD16/32) Antibody/ Biolegend/ 156604/ https://www.biolegend.com/it-it/products/trustain-fcx-plus-anti-mouse-cd16-32-antibody-17085?pdf=true&displayInline=true&leftRightMargin=15&topBottomMargin=15&filename=TruStain%20FcX%E2%84%A2%20PLUS%20(anti-mouse%20CD16/32)%20Antibody.pdf

Brilliant Violet 711™ anti-mouse CD45 Antibody/ Biolegend/ 103147/ https://www.biolegend.com/it-it/products/brilliant-violet-711-anti-mouse-cd45-antibody-10439?pdf=true&displayInline=true&leftRightMargin=15&topBottomMargin=15&filename=Brilliant%20Violet%20711%E2%84%A2%20anti-mouse%20CD45%20Antibody.pdf

APC/Cyanine7 anti-mouse Ly-6G Antibody/ BioLegend/ 127624/ https://www.biolegend.com/it-it/products/apc-cyanine7-anti-mouse-ly-6g-antibody-6755?pdf=true&displayInline=true&leftRightMargin=15&topBottomMargin=15&filename=APC/Cyanine7%20anti-mouse%20Ly-6G%20Antibody.pdf

CD11b Monoclonal Antibody (M1/70), PE, eBioscience™/ invitrogen _Thermo Fisher/ 12-0112-82/ https://www.thermofisher.com/order/genome-database/dataSheetPdf?producttype=antibody&productsubtype=antibody_primary&productId=12-0112-82&version=237

FITC Rat Anti-Mouse Ly-6C/ BD Biosciences/ 553104/ https://www.bdbiosciences.com/content/bdb/paths/generate-tds-document.ca.553104.pdf

Alexa Fluor® 647 Rat Anti-Mouse Siglec-F/ BD Bioscience/ 562680/ https://www.bdbiosciences.com/content/bdb/paths/generate-tds-document.ca.562680.pdf

F4/80 Monoclonal Antibody (BM8), PE-Cyanine7, eBioscience™ / invitrogen _ Thermo Fisher/ 25-4801-82/ https://www.thermofisher.com/order/genome-database/dataSheetPdf?producttype=antibody&productsubtype=antibody_primary&productId=25-4801-82&version=237

Alexa Fluor 488-coupled goat anti-rabbit / ThermoFisher Scientific / A11008 / https://www.thermofisher.com/antibody/product/Goat-anti-Rabbit-IgG-H-L-Cross-Adsorbed-Secondary-Antibody-Polyclonal/A-11008

Alexa Fluor 546-coupled goat anti-mouse / ThermoFisher Scientific / A-11003 / https://www.thermofisher.com/antibody/product/Goat-anti-Mouse-IgG-H-L-Cross-Adsorbed-Secondary-Antibody-Polyclonal/A-11003

CF 594-coupled goat anti-rat / Sigma-Aldrich / SAB4600111 / https://www.sigmaaldrich.com/DE/de/product/sigma/sab4600111

Eukaryotic cell lines

Policy information about cell lines

Cell line source(s)	HL-1 (SCC065, Sigma); HVF (T4038, Applied Biological Materials Inc.); THP-1 (88081201, Sigma)
Authentication	Cell lines used were not authenticated
Mycoplasma contamination	All cell lines, primary cells, stem cells and iPSC-derived cardiomyocytes were routinely tested negative for mycoplasma
Commonly misidentified lines (See ICLAC register)	No cell line is listed by ICLAC

Animals and other organisms

Policy information about studies involving animals; ARRIVE guidelines recommended for reporting animal research

Laboratory animals	8-week-old female wild-type C57BL/6J (ADAM10 Inhibitor experiments); 8-12-week-old female and male ADAM10 wild-type or knockout C57BL/6N (ADAM10 KO experiments)
Wild animals	None
Field-collected samples	None
Ethics oversight	Animal facilities and experiments were authorized by the Landesdirektion Dresden Germany, according to the German and Saxony animal welfare regulations (No. TVV 54/2016, No. TVV16/2022) and comply with the ARRIVE guidelines and the guidelines from Directive 2010/63/EU of the European Parliament on the protection of animals used for scientific purposes.

Note that full information on the approval of the study protocol must also be provided in the manuscript.

Human research participants

Policy information about studies involving human research participants

Population characteristics	Details on gender, age and clinical characteristics of these patients are published here: https://doi.org/10.1007/s00395-017-0635-0
Recruitment	Heart samples were obtained from 12 patients with NYHA stage III-IV. Non-failing myocardium originated from 6 healthy donor hearts that could not be transplanted for technical reasons. All participants gave written informed consent. The study was conducted in accordance with the Declaration of Helsinki. Self-selection bias cannot be completely excluded, since only patients who agreed and gave written consent have been included in the study. Number of inquired patients, who refuse to donate heart tissue for research purposes and potential reasons are not known. Thus self-selection bias can neither be estimated nor corrected.
Ethics oversight	Collection of human tissue samples was approved by the ethics committee of the University Medical Center Goettingen (Az.:31/9/00, this approval is also applicable for the acquisition of samples at the University Hospital Regensburg).

Note that full information on the approval of the study protocol must also be provided in the manuscript.

Flow Cytometry

Plots

Confirm that:

- [x] The axis labels state the marker and fluorochrome used (e.g. CD4-FITC).
- [x] The axis scales are clearly visible. Include numbers along axes only for bottom left plot of group (a 'group' is an analysis of identical markers).
- [x] All plots are contour plots with outliers or pseudocolor plots.
- [x] A numerical value for number of cells or percentage (with statistics) is provided.

Methodology

Sample preparation	Cells were prepared for single cell suspension from heart biopsies as outlined in the method section:<br><br>For analysis of leukocyte heart tissue infiltration, mouse hearts were perfused through the left ventricle with 5 mL ice-cold PBS, the infarct area dissected, minced with a fine scissor and digested in RPMI 1640 medium containing 1 mg/mL collagenase I (Roche) and 0.1mg/mL DNAse I (Roche) twice for 20 min at 37°C under agitation. Tissues were triturated and cells filtered through a 50-µm filter (CellTrics, Sysmex), washed, and centrifuged (5 min; 500g; 4°C). Approximately 5e+05 cells were used for analysis. First, cells were stained with Fc blocking antibody (1:200) for 10min on ice. Following cells were stained for 30 min at 4°C in PBS with FACS buffer (PBS -2% FBS – 2mM EDTA) with fluorochrome-conjugated antibodies. Prior flow cytometry, cells were washed twice and resuspended in FACS buffer. Data were acquired on an LSR Fortessa X-20 (BD Biosciences) and analyzed with the FlowJo software (BD).
Instrument	LSRFortessa X-20 (BD)
Software	Samples were acquired on the LSRFortessa X-20 (BD) with the BDFACS Diva software version 8.0.2. Flow cytometry data were analyzed using FlowJo version 10.8.0 (BD).
Cell population abundance	The murine heart contains at basal levels below 10% of leukocytes. However, upon myocardial infarction this population increases rapodly. On day 3 post MI, neutrophils are the most abundant leukocyte population, follwoed by the macrophage/moncyte subset.
Gating strategy	The gating strategy is outlined in Extended Data Fig. xx. Briefly, singlet cells were identified as (1) neutrophils (CD45+CD11b+Ly6G+), (2) eosinophils (CD45+CD11b+Ly6G-SigF+), (3) macrophages (CD45+CD11b+Ly6G- SigF- F4/80+ LY6C-) and (4) monocytes (CD45+CD11b+Ly6G-SigF-LY6C+). Initial FSC/SSC gate was kept broadly given that the analyzed immune cell population differ, depending on different levels of granularity and size. Populations were determined based on FMO (Full Minus One) controls.

- [x] Tick this box to confirm that a figure exemplifying the gating strategy is provided in the Supplementary Information.
